# Trends in Pediatric Candidemia: Epidemiology, Anti-Fungal Susceptibility, and Patient Characteristics in a Children’s Hospital

**DOI:** 10.3390/jof7020078

**Published:** 2021-01-22

**Authors:** Anabel Piqueras, Lakshmi Ganapathi, Jane F. Carpenter, Thomas Rubio, Thomas J. Sandora, Kelly B. Flett, Julia R. Köhler

**Affiliations:** 1 Pediatric Infectious Disease Unit, Pediatrics Department, University & Polytechnic Hospital La Fe, E-46026 Valencia, Spain; edupiqueras_ana@gva.es; 2 Division of Infectious Diseases, Boston Children’s Hospital and Harvard Medical School, Boston, MA 02115, USA; Lakshmi.Ganapathi@childrens.harvard.edu (L.G.); jane.carpenter@bmc.org (J.F.C.); Thomas.Sandora@childrens.harvard.edu (T.J.S.); kbflett@novanthealth.org (K.B.F.); 3 Lombardi Cancer Center, Georgetown University Hospital, Washington, DC 20007, USA; rubiott2000@yahoo.com

**Keywords:** *Candida*, bloodstream infection, pediatric, neonatal, anti-fungal

## Abstract

*Candida* bloodstream infections (CBSIs) have decreased among pediatric populations in the United States, but remain an important cause of morbidity and mortality. Species distributions and susceptibility patterns of CBSI isolates diverge widely between children and adults. The awareness of these patterns can inform clinical decision-making for empiric or pre-emptive therapy of children at risk for candidemia. CBSIs occurring from 2006–2016 among patients in a large children’s hospital were analyzed for age specific trends in incidence rate, risk factors for breakthrough-CBSI, and death, as well as underlying conditions. *Candida* species distributions and susceptibility patterns were evaluated in addition to the anti-fungal agent use. The overall incidence rate of CBSI among this complex patient population was 1.97/1000 patient-days. About half of CBSI episodes occurred in immunocompetent children and 14% in neonatal intensive care unit (NICU) patients. Anti-fungal resistance was minimal: 96.7% of isolates were fluconazole, 99% were micafungin, and all were amphotericin susceptible. Liposomal amphotericin was the most commonly prescribed anti-fungal agent included for NICU patients. Overall, CBSI-associated mortality was 13.7%; there were no deaths associated with CBSI among NICU patients after 2011. Pediatric CBSI characteristics differ substantially from those in adults. The improved management of underlying diseases and antimicrobial stewardship may further decrease morbidity and mortality from CBSI, while continuing to maintain low resistance rates among *Candida* isolates.

## 1. Introduction

*Candida* bloodstream infections (CBSIs) are a leading cause of invasive fungal infections in hospitalized children, and one of the most common causes of healthcare-associated infections worldwide. While the epidemiology of invasive fungal infections, including CBSI, varies according to the geographical region and patient population, globally non-*albicans Candida* species together cause the majority of CBSI, and resistant *Candida* species and strains are emerging among adult patient populations.

While the CBSI incidence in children has been decreasing in the United States over recent decades [1], as infection control and antimicrobial stewardship practices have continued to become more rigorous and universally implemented, CBSI still remain associated with high pathogen-related morbidity and mortality, increased length of hospitalization, and considerable resource utilization [2,3,4]. Some pediatric patient populations such as neonates and infants under the age of one, children with hematologic malignancies, and critically ill children in intensive care units are known to be at increased risk for CBSI, but established risk factors remain poor predictors of when and in whom CBSI occur [5]. Early diagnosis is especially challenging in children given the nonspecific symptoms of candidemia and the low sensitivity of blood cultures [6]. In some populations, such as premature neonates and neutropenic oncologic patients, empiric or pre-emptive anti-fungal therapy can decrease delays to the initiation of the appropriate therapy inevitably caused by the reliance on blood culture results.

In adults, active surveillance studies in two US East Coast cities have shown significant declines in candidemia incidence and in central venous catheter-associated candidemia between 2008 and 2013 [7]. In these studies, the predominant species were *C. albicans* (36%) and *C. glabrata* (27%) [7]. Older single-center studies comparing adult candidemia patients over the years 1983–1986, 1997–2001, and 2004–2007 showed decreasing percentages of *C. albicans* isolates from 61 to 47% over these periods and increasing percentages of *C. glabrata* (from 0 to 29%), while *C. parapsilosis* remained at 12% [8]. In the *Candida* isolates examined in that center over those periods, echinocandin resistance was not observed and the emergence of anti-fungal resistance was limited to 16% fluconazole resistant *C. glabrata* in the last study period [8]. However, pediatric and adult populations differ with respect to the prevalence of isolated *Candida* species and susceptibility patterns even in the same geographic areas [9,10]. Therefore, the surveillance of local species distribution and anti-fungal susceptibility patterns aids in the rational choice of empiric or pre-emptive therapy for children, in choices of prophylactic regimens, and in antimicrobial stewardship. The present study was undertaken to evaluate changes in epidemiology, anti-fungal susceptibility, patient characteristics, and management of CBSI in a large freestanding children’s hospital over an 11-year period.

## 2. Materials and Methods

The Boston Children’s Hospital (BCH) Institutional Review Board approved protocol IRB-P00024287, “Retrospective Review of *Candida* Bloodstream Infections,” on 11/30/2016. We then performed a retrospective cohort study of CBSI in patients admitted to BCH between January 2006 and December 2016. Patients were identified from the database of the BCH clinical microbiology laboratory.

### 2.1. Study Site

Boston Children’s Hospital (BCH) is a 406-bed quaternary care children’s hospital in Boston, Massachusetts with specialized units including neonatal, cardiac, medical, and medical/surgical intensive care units, as well as oncology, solid organ, and stem cell transplant units, which care for highly complex patient populations.

### 2.2. Definitions

A CBSI was defined as a positive blood culture for a *Candida* species alone. A CBSI episode was defined as a positive blood culture for only a *Candida* species ≥30 days before or after another blood culture growing *Candida* [11]. Polymicrobial blood cultures growing other organisms in addition to *Candida* species were not included. Breakthrough (BT)-CBSI was defined as a CBSI that occurred in patients receiving systemic anti-fungal agents for at least 3 days before the first positive blood culture [12]. Central line-associated blood stream infection (CLABSI) was defined according to the Centers for Disease Control and Prevention (CDC) surveillance criteria [13]. Recurrent CBSI was defined as a second or more episode of CBSI in the same patient, separated by at least 1 month. Patients were categorized as immunocompetent, immunocompromised, or in the neonatal intensive care unit (NICU). The latter group was considered separately due to the unique physiological immaturity of the neonatal immune system and skin barrier. Very preterm was defined as birth at less than 32 weeks of gestation and extremely preterm, defined as birth at or before 25 weeks of gestation. Mortality associated with CBSI was defined as death as a direct consequence of CBSI or death from CBSI-associated complications [3], maximally 60 days after the first positive culture as determined by two investigators upon chart review (A.I.P. and J.R.K.).

Primary *Candida* species identification was performed in the BCH microbiology laboratory. Blood samples were cultured in the BacT/AlerT 3D system (Biomérieux, Chicago, IL, USA). Positive culture bottles were plated on Sabouraud Dextrose Emmons (Becton Dickinson, Franklin Lakes, NJ, USA), in addition to bacteriologic agar media. Colonies growing on a Sabouraud medium were examined microscopically and yeast-shaped organisms were immediately inoculated to fetal bovine serum (Sigma Aldrich, St. Louis, MO, USA). Formation of germ tubes at 2.5–4 h confirmed speciation as *Candida albicans*. Isolates that did not produce germ tubes were speciated by the VITEK 2YST card (Biomérieux) and/or by the API20 C AuX (Biomérieux) strip and confirmed by morphology on a cornmeal agar medium. Anti-fungal susceptibility testing was performed at ARUP Laboratories, Salt Lake City, Utah, USA, a commercial clinical laboratory. In vitro susceptibility to micafungin, fluconazole, and voriconazole was defined by Clinical and Laboratory Standards Institute criteria, as well as using species-specific epidemiological cutoff values for less prevalent *Candida* species [14]. Multi-resistance was defined as resistance to two anti-fungal drug classes, azoles and echinocandins. 

A review of electronic medical records was performed on all patients within the study period with CBSI that met the study definitions. Relevant data collected from the electronic medical records included age, underlying disease, presence of neutropenia (neutrophil count <500/mm^3^), exposure to broad spectrum antibiotics (piperacillin-tazobactam, carbapenems, third and fourth generation cephalosporins, glycopeptides, aminoglycosides, and fluoroquinolones) in the 7 days preceding CBSI, exposure to systemic anti-fungals and steroids, and the anti-fungal agent used for the treatment when CBSI was identified. The presence of a central venous catheter (CVC) and receipt of parenteral nutrition (PN) at the time of CBSI were also recorded.

### 2.3. Outcome Measures and Statistical Analyses

Frequencies, percentages, and descriptive statistics were used to summarize patient characteristics and anti-fungal susceptibility overall, as well as by species of *Candida* causing CBSI. Differences in patient characteristics across species-specific CBSI were compared using the chi-square test of proportions for categorical variables and the Tukey’s multiple comparison test for distribution of the mean age of patients. The primary outcome was trends in the incidence rate of CBSI during the study period. Secondary outcomes included (1) age-specific trends in the incidence rate of CBSI, (2) risk factors for developing breakthrough-CBSI, and (3) risk factors associated with death. We calculated CBSI rates per 1000 patient days of hospitalization for each year in the study period, and *Candida* CLABSI rates per 1000 CVC days between 2011–2016, when data on CVC days became available. Additionally, we calculated annual CBSI rates per 1000 patient days by age group, using the following categories: Infants <1 year of age, children between ages of 1–4 years, and children >4 years of age. Annual trends in CBSI rates overall, *Candida* CLABSI rates, and CBSI rates by age group during the study period were evaluated using Poisson regression models. The secondary outcomes of breakthrough-CBSI and mortality were evaluated using multivariable logistic regression models. Odds ratios for relevant risk factors were first calculated using the univariate analysis and adjusted subsequently in multivariable models for age, gender, and other confounders. Risk factors in multivariable models were considered significant at a *p*-value of < 0.05. Analyses were conducted using SPSS version 26.0 (IBM, Armonk, New York, NY, USA).

## 3. Results

### 3.1. Characteristics of CBSI Episodes

**Figure 1:**
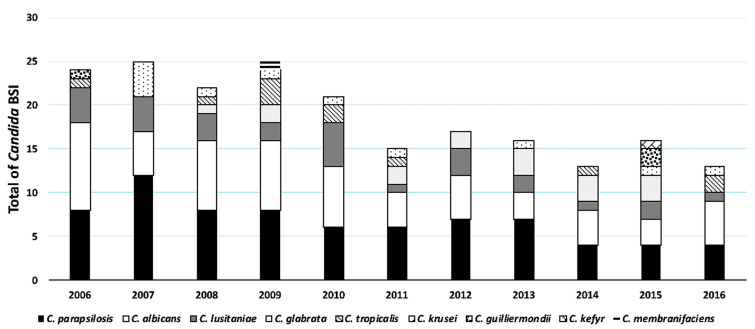
Number of *Candida* bloodstream infections (BSIs) per year, stratified by *Candida* species. Bloodstream infection episodes were tallied for each species and graphed for each year.

Between 2006–2016, there were 208 episodes of CBSI in 182 patients with an incidence rate of 1.97 episodes per 1000 patient-days. All episodes manifested >48 h after hospital admission and hence met the definition of being hospital-acquired. The mean age of patients overall was 6.8 years (Table 1). Infants <1 year of age and children between the age of 1–4 years had the highest CBSI incidence rates (1.77 and 2.58/1000 patient-days, respectively). Approximately half of the CBSI episodes occurred in immunocompetent patients, while about a third occurred in immunocompromised patients, with the remainder occurring in neonates in the NICU (Table 1). The distribution of *Candida* species causing CBSI in each year is displayed in Figure 1. Overall, *Candida parapsilosis* was the predominant species (35.6%), followed by *C. albicans* (29.8%). *C. lusitaniae* (13.4%) was the third most prevalent species isolated surpassing *C. glabrata* (7.7%). Patients with *C. glabrata* BSI were significantly older than patients with *C. parapsilosis* (*p* = 0.015) and patients with *C. lusitaniae* BSI (*p* = 0.004) (Table 1).

Three patients with *C. albicans* fungemia were co-infected with another *Candida* species: Two with *C. parapsilosis* and one with *C. glabrata*. In two of these patients, isolation of the distinct species was separated by one or 21 days, respectively. In one patient, both species were isolated from the same culture bottle.

Thirteen patients (7.1%) had 22 episodes of recurrent CBSI (Table 2). The subsequent episodes were caused by the same species in eight instances and by a different species in fourteen. All the episodes of recurrent CBSI occurred in medically complex patients with CVCs, receiving chronic parenteral nutrition. The predominant species in recurrent CBSI were *C. albicans*, *C. parapsilosis,* and *C. glabrata.*

### 3.2. Anti-Fungal Susceptibility

Susceptibility testing was performed for Amphotericin B (AmB) in 115 isolates, for caspofungin and voriconazole in 114 isolates, for micafungin in 89 isolates, and for fluconazole in 112 isolates (Table 3). All tested isolates were susceptible to AmB and voriconazole. Micafungin and caspofungin showed an excellent activity against most *Candida* species, including all *C. parapsilosis* isolates. Almost all *Candida* species isolates were susceptible to fluconazole (96.7%). Micafungin resistance was noted in two *C. glabrata* isolates, in a patient with prior episodes of CBSI treated with micafungin (Table 4). Multi-resistance was not found in any of the tested isolates.

### 3.3. Trends in the Prescription of Anti-Fungal Therapy

Overall, liposomal AmB was the most frequently prescribed anti-fungal agent, used particularly in neonates and empirically in neutropenic patients until central nervous system involvement was excluded. Micafungin use increased significantly between the first and second half of the study period (Supplementary Figure S1). Among neonates at our center treated for CBSI in the study period, only one patient received AmB deoxycholate for 5 days of a 28-day treatment course in the first studied year, 2006. All other AmB was administered as the liposomal formulation (amBisome) in this period.

### 3.4. Trends in the Incidence of CBSI

**Figure 2:**
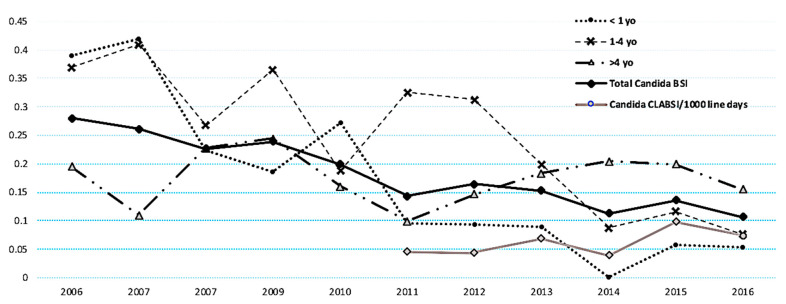
Annual *Candida* bloodstream infection rates. Overall annual CBSI rates (cases of CBSI/1000 patient days), by age group (2006–2016) and rate of *Candida* CLABSI (CLABSI/1000 line days) from 2011 to 2016. Incidence rate ratio (IRR) of total *Candida* BSI: 0.84 (95% CI: 0.81–0.88), *p* < 0.001; IRR of *Candida* BSI for <1 year olds: 0.78 (95% CI 0.71–0.85), *p* < 0.001; IRR of *Candida* BSI for 1–4 year olds: 0.87 (95% CI: 0.80–0.95), *p* = 0.002; IRR of *Candida* BSI for >4 year olds: 1.00 (95% CI 0.94–1.06), *p* = 0.96. IRR of *Candida* CLABSI: 0.87 (95% CI: 0.70–1.09), *p* = 0.22.

During the study period, there was a 16% decrease per year in the annual incidence rate of CBSI (incidence rate ratio (IRR): 0.84, 95% confidence interval (CI) 0.81–0.88, *p* < 0.001) (Figure 2). Infants <1 year of age and children between the age of 1–4 years had the highest CBSI incidence rates overall (1.77 and 2.58/1000 patient-days, respectively). The analysis of age-specific trends in CBSI demonstrated a decline in infants aged <1 year (IRR 0.78, 95% CI 0.71–0.85, *p* < 0.001) and children aged 1–4 years (IRR 0.87, 95% CI 0.80–0.95, *p* = 0.002). There was no decline in the annual incidence of CBSI in patients older than 4 years (IRR 1.00, 95% CI 0.94–1.06, *p* = 0.96) (Figure 2). Similarly, there was no decline in the annual incidence of *Candida* CLABSI between 2011–2016, the years for which CLABSI data were available (IRR 0.87, 95% CI: 0.70–1.09, *p* = 0.22).

### 3.5. Risk Factors for Breakthrough CBSI

BT-CBSI accounted for 17% of the CBSI (36 cases, four of them recurrent episodes) and did not increase over the study period. The most common infecting species in BT-CBSI was *C. parapsilosis*, followed (36%) by *C. albicans* (19.4%), *C. krusei* (14%), and *C. glabrata* (11%). Anti-fungal agents administered at the time of BT-CBSI and the species isolated are shown in Supplementary Figure S2. Once BT-CBSI was suspected, a combination therapy was chosen in 12 cases, AmB in 21 cases, and micafungin in three cases. All the isolates were susceptible to prior prophylactic anti-fungals, as well as the empiric anti-fungal agents chosen. Risk factors significantly associated with BT-CBSI included underlying immunosuppression and exposure to broad-spectrum antibiotics (Table 5).

### 3.6. Risk Factors for Mortality

The annual mortality due to CBSI did not change during the study period. The overall 30-day mortality rate was 13.7% with a median time-to-death of 6 days (range, 1–60) from the onset of CBSI. Among the patients who expired, 11 were immunocompromised, seven were immunocompetent, and seven were neonates. All neonatal deaths attributed to CBSI were prior to 2012, three of which occurred in extremely preterm infants. Risk factors independently associated with death included exposure to systemic steroids and BT-CBSI (Table 6).

## 4. Discussion

A number of pediatric studies have addressed specific aspects of the epidemiology, species distribution, anti-fungal susceptibility profiles, and treatment of CBSI, as well as outcome-associated factors [2,4,12,15,16,17,18]. In our study, we reviewed these issues together from the neonatal period until young adulthood over more than a decade, providing an integrated view of trends in this infection.

The overall incidence rate of CBSI at this institution decreased by 62% over the 11-year period, consistent with similar trends reported by others [7,19,20,21]. The downward trend in CBSI predominated in younger children. Moreover, Cleveland et al. reported a decline in the crude incidence rate of CBSI in infants aged <1 year in the past two decades [11].

Anti-fungal prophylaxis cannot explain the decline in incidence since routine anti-fungal prophylaxis in this pediatric hospital was limited to patients with cancer. We speculate that the observed CBSI decrease is multifactorial and includes a temporal trend of improved management of underlying diseases in surgery, oncology, and neonatology, as well as antimicrobial stewardship efforts. Since a *Candida* source is typically endogenous and may be less impacted by some infection control measures (e.g., healthcare worker hand hygiene or isolation precautions), preserving a balanced microbiome by reducing broad-spectrum antibiotic exposure whenever possible will likely favorably impact the CBSI risk.

Recent studies showed significant decreases in overall and *Candida*-specific CLABSI [22,23,24,25]. In our series, the rate of *Candida* CLABSI did not decrease in the observed half-decade for which CVC data were available, though the small annual sample sizes limit further conclusions. Measures to improve CVC care may affect *Candida* CLABSI less than those caused by skin flora, given that they frequently arise from gastrointestinal translocation [7]. Alternatively, achievable effects of rigorous catheter care and infection prevention may already have been maximized at the beginning of this half-decade. Similar to the others, we did not find a predominance of immunocompromised patients in CBSI [4,16]. However, a large majority of patients had chronic diseases and CVC.

Similar to the others, we found that non-*albicans Candida* species, particularly *C. parapsilosis*, now predominate in pediatric BSI [4,15,16,26,27]. This contrasts with species distributions in adult patients. A recent survey of electronic medical records from 203 hospitals across the United States covering the years 2009–2017 for all patient age groups, identified 18,728 invasive candidiasis cases of 16,334 patients (among which 9839 cases represented candidemia) using microbiology laboratory data, as we did. Forty percent of the patients were >65 years old [10], while 864 (5%) were <18 years of age [10], suggesting that the findings of this large study are representative of adult candidiasis cases across the United States. Candidiasis incidence in this study was calculated by hospitalizations and not by patient years, as we did. Hence, a direct comparison with our results is not possible, but the downward trend of candidemia observed over the years of this study was not significant [10]. While *Candida* species distributions were not shown specifically for candidemia, overall 48% of isolates were *C. albicans*, 24% *C. glabrata*, 11% *C. parapsilosis*, 7% *C. tropicalis,* and 6% of isolates represented 26 other non-albicans species including *C. lusitaniae* (10)*,* highlighting the starkly different species distribution in our pediatric populations. Distinct host characteristics, less use of azole prophylaxis, and preferential administration of AmB over fluconazole to treat childhood CBSI may explain the discrepancy in *Candida* species between adult and pediatric series [28]. Liposomal AmB is tolerated in children much better than in adults, and its frequent use in our population may have limited emergence of anti-fungal-resistant species and strains, given the fitness defects of amphotericin-resistant *Candida* [29].

Of note, *C. lusitaniae* was the third most common *Candida* species causing BSI in our institution across the age groups. This species has rarely been reported previously as a cause of BSI and accounts for 1–2% of all non-*albicans Candida* BSI across multiple studies [4,15,16,18,26,28,30,31,32]. The use of polyenes has been associated with the selection of this species [33], which can develop resistance to AmB during therapy. AmB monotherapy has been associated with a poor response especially in immunocompromised patients [32,34]. In our series, all tested *C. lusitaniae* were susceptible to AmB, though susceptibilities were not obtained in 32% of the isolates. *C. lusitaniae* has also been reported to be resistant to fluconazole, as two of our cases were. Therefore, anti-fungal susceptibilities of *C. lusitaniae* isolates should be routinely tested, and recalcitrant infections should be re-examined for the development of resistance.

Anti-fungal resistance rates were extremely low in our study, with no signs of resistance emergence over the 11-year period, possibly related to the infrequent use of azole prophylaxis among our patients. Micafungin showed excellent activity against almost all *Candida* isolates including *C. parapsilosis*. Similar to other studies [35], we found all *C. parapsilosis* isolates to be susceptible to micafungin, and its use was not associated with clinical failure. 

In children, mortality rates associated with CBSI have been reported between 9.3% and 37% [2,3,4,15,17,26]. Heterogeneity among studies, centers, and patient populations may explain the wide range of mortality in the literature. Mortality due to CBSI may be difficult to differentiate from that caused by the underlying illness even with propensity score analysis [2,36,37]. Consistent with other reports [30,38], mortality in our institution did not improve over the 11-year period and was not related to anti-fungal resistance.

However, since 2012, CBSI-related mortality did not occur in children ≤4 years in our study. A low threshold for empiric AmB use in infants in our institution may have accounted for the improved prognosis in younger patients. AmB resistance in most *Candida* species is extremely rare despite five decades of use. Moreover, strains that evolve AmB resistance exhibit diminished fitness and are less virulent [29].

Notably, our experience differs from the findings of Ascher et al. [39] that in neonates, lipid AmB formulations were associated with a higher mortality than the deoxycholate formulation. In that study, no distinction was made between three available lipid formulations of AmB, while in our hospital, only liposomal AmB (Ambisome) is used. The use of different liposomal amphotericin formulations between the neonatology centers included in the Ascher et al. study, in addition to specific center characteristics such as the prevalence of extreme prematurity, also may have influenced the observed mortality. Our findings support the use of liposomal AmB in neonates.

Our study has several important limitations. As a retrospective study from a single quaternary care pediatric hospital, the results may not be generalizable to children receiving care in other settings. Residual confounding from unmeasured factors, including severity of illness, is likely present and may influence associations between CBSI and mortality. More generally, identifying candidemia from blood culture data inevitably leads to a very significant undercount, given the low sensitivity of blood cultures for this infection of <50% [40,41]. This undercount currently affects all similar studies, while in the future, the routine use of next-generation sequencing as a molecular diagnostics tool could overcome this limitation.

In summary, we found a decreasing incidence rate of CBSI over time. *C. parapsilosis* and *C. albicans* BSI decreased, and CBSI-related mortality was absent after 2011 in younger children, resulting in an increased median age for CBSI and fatal cases over the 11-year period. Anti-fungal resistance was very low and did not increase over time. Microbiologic trends in pediatric CBSI differ from those in adults possibly since AmB use is rarely limited by toxicities in children. Further decreasing CBSI and improving their outcomes may require further improvements in the management of underlying comorbidities, including prematurity and malignancies, as well as improved diagnostics that permit treatment earlier in the infection course.

## Figures and Tables

**Table 1:** Clinical characteristics and outcome of patients with *Candida* BSI.

	All *Candida* spp.	*C. parapsilosis*	*C. albicans*	*C. lusitaniae*	*C. glabrata*	*C. krusei*	*C. tropicalis*	Other Species
No. of episodes (%)	208	74 (35.6)	62 (29.8)	28 (13.4)	16 (7.7)	11 (5.3)	11 (5.3)	6 (2.9)
No. of Patients	182	71	56	28	14	11	11	6
Males (%)	91 (50.0)	41 (57.7)	29 (51.8)	13 (46.4)	6 (42.9)	6 (54.4)	3 (27.3)	3 (50.0)
Age, mean [SD]	6.8 [7.9]	6.0 [7.2]	7.6 [7.4]	4.1 [7.0]	12.7 [9.7]	9.9 [7.6]	4.5 [4.5]	4.9 [5.3]
<1 year (%)	49 (23.6)	20 (27.0)	14 (22.6)	11 (39.3)	1 (6.3)	0 (0.0)	1 (9.1)	2 (33.3)
1–4 years (%)	56 (26.9)	21 (28.4)	13 (21.0)	9 (32.1)	4 (25.0)	1 (9.1)	6 (54.5)	2 (33.3)
>4 years (%)	103 (49.5)	33 (44.6)	35 (56.5)	8 (28.6)	11 (68.8)	10 (90.9)	4 (36.4)	2 (33.3)
**Immunocompetent**	106 *	**35**	**32**	**14**	**13**	**3**	**8**	**1**
Gastrointestinal disorder	70	22	22	8	9	3	5	1
Cardiopulmonary disorder	29	10	7	5	5	0	2	1
Neurological disorder	16	8	5	2	0	0	1	0
Renal/urological disorder	10	4	2	2	1	0	1	0
Other underlying disease **	9	5	3	0	0	1	0	0
**Immunocompromised**	**73**	**25**	**21**	**9**	**3**	**8**	**3**	**4**
Bone marrow transplant	13	4	3	2	1	3	0	0
Solid organ transplant	10	1	5	1	1	1	0	1
Oncology	35	12	11	5	1	2	2	2
Other immunodeficiencies	15	8	2	1	0	2	1	1
**NICU**	**29**	**14**	**9**	**5**	**0**	**0**	**0**	**1**
Risk factors								
Neutropenia	29	5	9	4	2	4	2	3
Antibiotic exposure	129	46	40	15	10	9	6	3
Anti-fungal prophylaxis	36	13	7	4	4	5	0	3
Systemic steroids exposure	53	22	17	5	3	3	2	1
Central venous catheter	195	68	55	28	16	11	11	5
Parenteral nutrition	131	47	37	17	10	10	8	2
Mortality *n* (%)	25 (13.7)	9 (12.7)	5 (8.9)	3 (10.7)	2 (14.3)	3 (27.3)	2 (18.2)	1 (16.7)

Other species included *C. guilliermondii* (3), *C. kefyr* (1), *C. famata* (1), and *C. membranifaciens* (1). * Twenty-eight patients had more than one underlying disorder; ** mitochondrial dysfunction (5), dystrophic epidermolysis bullosa (3), chromosomal disorder (1).

**Table 2:** *Candida* species distribution in 13 patients with recurrent CBSI and anti-fungals received in previous episodes.

*Candida* species-	First Episode	Recurrent Episodes	Anti-Fungals Received in Previous Episodes		
			Amphotericin B	Micafungin	Fluconazole
*C. parapsilosis*	4	6	3		1
*C. albicans*	5	8	6	2	1
*C. lusitaniae*	3	1	1	1	
*C. glabrata*		6		6	
*C. krusei*		1	1	2	1
*C. tropicalis*	1				

**Table 3:** In vitro susceptibilities of *Candida* BSI.

BSI								
Species	Drug	MIC (mg/L)			ECV (mg/L) ^a^	Susceptibility		
		MIC Range	MIC_50_	MIC_90_		S	I/SDD	R
*C. albicans* (31)	AMB	0.25–1	0.5	0.5	1	31	-	-
	CAS	0.016–0.5	0.031	0.125	0.25	30	1	-
	MCF	≤0.008–0.03	≤0.008	0.016	0.06	22	-	-
	FZ	0.12–1	0.25	0.5	1	31	-	-
	VOR	≤0.008–0.03	≤0.008	0.016	0.016	31	-	-
*C. parapsilosis* (45)	AMB	0.25–1	0.5	0.5	1	45	-	-
	CAS	0.125–1	0.25	0.5	2	44	-	-
	MCF	0.25–2	1	1	4	32	-	-
	FZ	0.25–8	1	2	2	41	3	1
	VOR	≤0.008–0.06	≤0.016	0.03	0.016	44	-	-
*C. glabrata* (11)	AMB	0.5–1	0.5	1	1	11	-	-
	CAS	0.016–1	0.06	0.25	0.25	9	1	1
	MCF	≤0.008 – 0.016	0.016	0.25	0.03	9	-	2
	FZ	2–64	4	16	64	10	-	1
	VOR	0.016–1	0.06	0.5	2	11	-	-
*C. krusei* (7)	AMB	0.5–1	1	1	128	6	-	-
	CAS	0.125–0.25	0.25	0.25	1	7	-	-
	MCF	0.06–0.125	0.06	0.125	0.25	5	-	-
	VOR	0.06–0.5	0.25	0.25	0.25	7	-	-
*C. tropicalis* (6)	AMB	0.25–1	0.5	1	1	6	-	-
	CAS	0.016–0.06	0.03	0.06	0.25	6	-	-
	MCF	0.016–0.03	0.03	0.03	0.06	4	-	-
	FZ	0.5–2	1	1	2	6	-	-
	VOR	0.03–0.125	0.06	0.125	0.5	6	-	-
Other spp. ^b^ (22)	AMB	0.125–1	0.25	0.5	NA	16	-	-
	CAS	0.016–1	0.25	1	NA	22	-	-
	MCF	0.03–1	0.06	0.125	NA	16	-	-
	FZ	0.25–32	1	4	NA	20	-	2
	VOR	≤0.008–0.125	0.016	0.125	NA	22	-	-
All spp. (122)	AMB	0.125–1	0.5	1,0	NA	115	-	-
	CAS	0.016–1	0.25	0.5	NA	121	1	1
	MCF	≤0.008–2	0.06	1	NA	89	-	2
	FZ	0.12–64	1	4	NA	115	3	4
	VOR	≤0.008–0.125	0.016	0.12	NA	121	-	-

AMB: Amphotericin B; CASL: Caspofungin; MCF: Micafungin; FZ: Fluconazole; VOR: Voriconazole; NWT: Non-wild type; S: Susceptible; SDD: Susceptible dose dependent; I: Intermediate; R: Resistant; ^a^ ECVs: Epidemiological cutoff values for 97.5% of the population, based on MICs obtained by SYO; ^b^ other species include *C. lusitaniae* (*n =* 19); *C. guilliermondii* (*n =* 2); and *C. famata* (*n =* 1).

**Table 4:** Clinical characteristics and minimum inhibitory concentrations (MIC) of patients with resistant species during the study period.

No.	Year	Age	CBSI Species	Underlying Condition	Anti-fungal Prophylaxis	Antibiotics Exposure	Fluconazole	Caspofungin	Micafungin
							MIC (mg/L)		
1	2006	3 years	*C. parapsilosis*	Short bowel syndrome, TPN	no	yes	**8**	1	-
2	2007	3 years	*C. albicans*	Heart transplant	no	yes	≤0.12	**0.5**	-
3	2007	5 months	*C. lusitaniae*	Short bowel syndrome, TPN	no	no	**32**	0.5	-
5	2013	8 years	*C. glabrata*	Intestinal failure, megabladder, TPN	no	no	2	0.25	**0.25**
6	2014	9 years	*C. glabrata*	Intestinal failure, megabladder, TPN	Amphotericin B	no	4	**0.5**	**1**
7	2014	21 years	*C. glabrata*	Cystic fibrosis, lung transplant	Voriconazole	yes	**64**	0.03	0.016
8	2015	7 years	*C. lusitaniae*	Cystic fibrosis	no	yes	**16**	0.12	0.06

TPN: Total parenteral nutrition; numbers 5 and 6 correspond to the same patient. In bold, resistant strains. For the C. *albicans* strain, in bold, intermediate susceptibility.

**Table 5:** Risk factors associated with breakthrough Candida blood stream infections.

Risk Factors	Odds Ratio [95% CI]	Adjusted Odds Ratio [95% CI]
***Candida* species**		
*Non-albicans* species	1.95 [0.80–4.72]	2.15 [0.82–5.69]
*C. albicans*	--	Ref
**Immune status**		
Immunocompromised	5.64 [2.35–13.5]	3.20 [1.02–10.01] **
Neonate	2.55 [0.77–8.50]	3.54 [0.89–14.11]
Immunocompetent	--	Ref
**Neutropenia**		
Yes	5.51 [2.35–12.9]	2.21 [0.73–6.67]
No	--	Ref
**Previous steroids**		
Yes	3.38 [1.60–7.16]	1.34 [ 0.53–3.38]
No	--	Ref
**Previous antibiotics**		
Yes	6.19 [2.10–18.3]	4.83 [1.54–15.20] ***
No	--	Ref
**Parenteral nutrition**		
Yes	1.22 [0.57–2.59]	2.07 [0.82–5.21]
No	--	Ref
**Central line access**		
Yes	2.63 [0.33–20.9]	0.81 [0.08–7.89]
No	--	Ref

Multivariable models adjusted for age and gender. ** *p* < 0.05, *** *p* < 0.01.

**Table 6:** Risk factors associated with death.

Risk Factors	Odds Ratio [95% CI]	Adjusted Odds Ratio [95% CI]
***Candida species***		
*Non-albicans* species	1.82 [0.65–5.10]	1.85 [0.61–5.62]
*C. albicans*	--	Ref
**Immune status**		
Immunocompromised	2.55 [0.94–6.93]	1.01 [0.23–4.41]
Immunocompetent	--	Ref
**Neutropenia**		
Yes	1.73 [0.59–5.05]	0.67 [0.21–2.20]
No	--	Ref
**Breakthrough Candidemia**		
Yes	4.93 [2.02–12.1]	3.60 [1.35–9.57] **
No	--	Ref
**Previous Steriods**		
Yes	4.82 [2.03–11.5]	3.83 [1.49–9.83] ***
No	--	Ref
**Gender**		
Male	0.47 [0.20–1.13]	0.43 [0.17–1.11]
Female	--	Ref
***Age***	--	1.04 [0.99–1.10]

** *p* < 0.05; *** *p* < 0.01.

## Data Availability

Not applicable.

## Supplementary Materials

The following are available online at https://www.mdpi.com/2309-608X/7/2/78/s1. Figure S1: Anti-fungal agents used for specific *Candida* species; Figure S2: Anti-fungals received at the time of breakthrough CBSI and species causing breakthrough.

## References

[1] Ota K.V., McGowan K.L. (2012). Declining incidence of candidemia in a tertiary inpatient pediatric population. J. Clin. Microbiol..

[2] Zaoutis T.E., Argon J., Chu J., Berlin J.A., Walsh T.J., Feudtner C. (2005). The epidemiology and attributable outcomes of candidemia in adults and children hospitalized in the United States: A propensity analysis. Clin. Infect. Dis..

[3] Tsai M.H., Wang S.H., Hsu J.F., Lin L.C., Chu S.M., Huang H.R., Chiang M.C., Fu R.H., Lu J.J., Huang Y.C. (2015). Clinical and molecular characteristics of bloodstream infections caused by Candida albicans in children from 2003 to 2011. Clin. Microbiol. Infect..

[4] Chan S., Baley E.D., Hossain J.D., Pentima M.C. (2015). Candida species bloodstream infections in hospitalised children: A 10-year experience. J. Paediatr. Child Health.

[5] Fisher B.T., Ross R.K., Roilides E., Palazzi D.L., Abzug M.J., Hoffman J.A., Berman D.M., Prasad P.A., Localio A.R., Steinbach W.J. (2016). Failure to validate a multivariable clinical prediction model to identify pediatric intensive care unit patients at high risk for candidemia. J. Pediatr. Infect. Dis. Soc..

[6] Clancy C.J., Nguyen M.H. (2013). Finding the “missing 50%” of invasive candidiasis: How nonculture diagnostics will improve understanding of disease spectrum and transform patient care. Clin. Infect. Dis..

[7] Cleveland A.A., Harrison L.H., Farley M.M., Hollick R., Stein B., Chiller T.M., Lockhart S.R., Park B.J. (2015). Declining incidence of candidemia and the shifting epidemiology of Candida resistance in two US metropolitan areas, 2008–2013: Results from population-based surveillance. PLoS ONE.

[8] Diekema D., Arbefeville S., Boyken L., Kroeger J., Pfaller M. (2012). The changing epidemiology of healthcare-associated candidemia over three decades. Diagn. Microbiol. Infect. Dis..

[9] Lamoth F., Lockhart S.R., Berkow E.L., Calandra T. (2018). Changes in the epidemiological landscape of invasive candidiasis. J. Antimicrob. Chemother..

[10] Ricotta E.E., Lai Y.L., Babiker A., Strich J.R., Kadri S.S., Lionakis M.S., Prevots D.R., Adjemian J. (2020). Invasive candidiasis species distribution and trends, United States, 2009–2017. J. Infect. Dis..

[11] Cleveland A.A., Farley M.M., Harrison L.H., Stein B., Hollick R., Lockhart S.R., Magill S.S., Derado G., Park B.J., Chiller T.M. (2012). Changes in incidence and antifungal drug resistance in candidemia: Results from population-based laboratory surveillance in Atlanta and Baltimore, 2008–2011. Clin. Infect. Dis..

[12] Lai M.Y., Hsu J.F., Chu S.M., Wu I.H., Huang H.R., Lin C.C., Lee I.T., Chiang M.C., Fu R.H., Tsai M.H. (2017). Breakthrough candidemia in children: Clinical and microbiological characteristics, therapeutic strategies and impact on outcomes. Future Microbiol..

[13] Prevention CfDCa (2017). Bloodstream Infection Event (Central Line-Associated Bloodstream Infection and Non-Central Line-Associated Bloodstream Infection). https://www.cdc.gov/nhsn/pdfs/pscmanual/4psc_clabscurrent.pdf.

[14] Pfaller M.A., Diekema D.J. (2012). Progress in antifungal susceptibility testing of Candida spp. by use of Clinical and Laboratory Standards Institute broth microdilution methods, 2010 to 2012. J. Clin. Microbiol..

[15] Celebi S., Hacimustafaoglu M., Ozdemir O., Ozkaya G. (2008). Nosocomial candidaemia in children: Results of a 9-year study. Mycoses.

[16] Neu N., Malik M., Lunding A., Whittier S., Alba L., Kubin C., Saiman L. (2009). Epidemiology of candidemia at a Children’s hospital, 2002 to 2006. Pediatr. Infect. Dis. J..

[17] Dutta A., Palazzi D.L. (2011). Candida non-albicans versus Candida albicans fungemia in the non-neonatal pediatric population. Pediatr. Infect. Dis. J..

[18] Warris A., Pana Z.D., Oletto A., Lundin R., Castagnola E., Lehrnbecher T., Groll A.H., Roilides E., Andersen C.T., Arendrup M.C. (2020). Etiology and outcome of candidemia in neonates and children in Europe: An 11-year multinational retrospective study. Pediatr. Infect. Dis. J..

[19] Fridkin S.K., Kaufman D., Edwards J.R., Shetty S., Horan T. (2006). Changing incidence of Candida bloodstream infections among NICU patients in the United States: 1995–2004. Pediatrics.

[20] Strollo S., Lionakis M.S., Adjemian J., Steiner C.A., Prevots D.R. (2016). Epidemiology of hospitalizations associated with invasive candidiasis, United States, 2002–2012(1). Emerg. Infect. Dis..

[21] Caggiano G., Lovero G., Giglio D.O., Barbuti G., Montagna O., Laforgia N., Montagna M.T. (2017). Candidemia in the Neonatal Intensive Care Unit: A Retrospective, Observational Survey and Analysis of Literature Data. Biomed. Res. Int..

[22] Miller M.R., Griswold M., Harris J.M., Yenokyan G., Huskins W.C., Moss M., Rice T.B., Ridling D., Campbell D., Margolis P. (2010). Decreasing PICU catheter-associated bloodstream infections: NACHRI’s quality transformation efforts. Pediatrics.

[23] Huskins W.C. (2012). Quality improvement interventions to prevent healthcare-associated infections in neonates and children. Curr. Opin. Pediatr..

[24] Li L., Fortin E., Tremblay C., Ngenda-Muadi M., Quach C. (2016). Central-line-associated bloodstream infections in quebec Intensive Care Units: Results from the provincial healthcare-associated infections surveillance program (SPIN). Infect. Control. Hosp. Epidemiol..

[25] Dandoy C.E., Hausfeld J., Flesch L., Hawkins D., Demmel K., Best D., Osterkamp E., Bracke T., Nagarajan R., Jodele S. (2016). Rapid cycle development of a multifactorial intervention achieved sustained reductions in central line-associated bloodstream infections in haematology oncology units at a children’s hospital: A time series analysis. BMJ Qual. Saf..

[26] Levy I., Rubin L.G., Vasishtha S., Tucci V., Sood S.K. (1998). Emergence of Candida parapsilosis as the predominant species causing candidemia in children. Clin. Infect. Dis..

[27] Garcia-Rodriguez J., Canton E., Peman J., Alvarez M., Ezpeleta G., Gomez-Nieto A., Iglesias I., Martin-Mazuelos E., Ramirez-de Ocariz I., Rezusta A. (2013). Age group, geographical incidence and patterns of antifungal susceptibility of Candida species causing candidemia in the Spanish paediatric population. Enferm. Infecc. Microbiol. Clin..

[28] Pfaller M.A., Diekema D.J., Jones R.N., Messer S.A., Hollis R.J. (2002). Trends in antifungal susceptibility of Candida spp. isolated from pediatric and adult patients with bloodstream infections: SENTRY Antimicrobial Surveillance Program, 1997 to 2000. J. Clin. Microbiol..

[29] Vincent B.M., Lancaster A.K., Scherz-Shouval R., Whitesell L., Lindquist S. (2013). Fitness trade-offs restrict the evolution of resistance to amphotericin B. PLoS Biol..

[30] Abelson J.A., Moore T., Bruckner D., Deville J., Nielsen K. (2005). Frequency of fungemia in hospitalized pediatric inpatients over 11 years at a tertiary care institution. Pediatrics.

[31] Wisplinghoff H., Ebbers J., Geurtz L., Stefanik D., Major Y., Edmond M.B., Wenzel R.P., Seifert H. (2014). Nosocomial bloodstream infections due to Candida spp. in the USA: Species distribution, clinical features and antifungal susceptibilities. Int. J. Antimicrob. Agents.

[32] Pfaller M.A., Jones R.N., Castanheira M. (2014). Regional data analysis of Candida non-albicans strains collected in United States medical sites over a 6-year period, 2006–2011. Mycoses.

[33] Krcmery V., Barnes A.J. (2002). Non-albicans Candida spp. causing fungaemia: Pathogenicity and antifungal resistance. J. Hosp. Infect..

[34] Minari A., Hachem R., Raad I. (2001). Candida lusitaniae: A cause of breakthrough fungemia in cancer patients. Clin. Infect. Dis..

[35] Fernández-Ruiz M., Aguado J.M., Almirante B., Lora-Pablos D., Padilla B., Puig-Asensio M., Montejo M., García-Rodríguez J., Pemán J., Ruiz P.d.P.M. (2014). Initial use of echinocandins does not negatively influence outcome in Candida parapsilosis bloodstream infection: A propensity score analysis. Clin. Infect. Dis..

[36] Gudlaugsson O., Gillespie S., Lee K., Vande B.J., Hu J., Messer S., Herwaldt L., Pfaller M., Diekema D. (2003). Attributable mortality of nosocomial candidemia, revisited. Clin. Infect. Dis..

[37] Falagas M.E., Apostolou K.E., Pappas V.D. (2006). Attributable mortality of candidemia: A systematic review of matched cohort and case-control studies. Eur. J. Clin. Microbiol. Infect. Dis..

[38] Bassetti M., Merelli M., Righi E., Diaz-Martin A., Rosello E.M., Luzzati R., Parra A., Trecarichi E.M., Sanguinetti M., Posteraro B. (2013). Epidemiology, species distribution, antifungal susceptibility, and outcome of candidemia across five sites in Italy and Spain. J. Clin. Microbiol..

[39] Ascher S.B., Smith P.B., Watt K., Benjamin D.K., Cohen-Wolkowiez M., Clark R.H., Benjamin D.K.J., Moran C. (2012). Antifungal therapy and outcomes in infants with invasive Candida infections. Pediatr. Infect. Dis. J..

[40] Lewis R.E., Cahyame-Zuniga L., Leventakos K., Chamilos G., Ben-Ami R., Tamboli P., Tarrand J., Bodey G.P., Luna M., Kontoyiannis D.P. (2013). Epidemiology and sites of involvement of invasive fungal infections in patients with haematological malignancies: A 20-year autopsy study. Mycoses.

[41] Clancy C.J., Nguyen M.H. (2018). Diagnosing Invasive Candidiasis. J. Clin. Microbiol..

