# Supplementary material for: Trends in Pediatric Candidemia: Epidemiology, Anti-Fungal Susceptibility, and Patient Characteristics in a Children’s Hospital
Source: J Fungi (Basel). 2021 Jan 22;7(2):78. doi: 10.3390/jof7020078 (PMC7911199; doi:10.3390/jof7020078)
Supplement: Supplementary file 1 [file jof-07-00078-s001.pdf]

Journal of Fungi

MDPI

Supplementary Materials: Trends in Pediatric Candidemia: Epidemiology, Anti-Fungal Susceptibility, and Patient Characteristics in a Children's Hospital

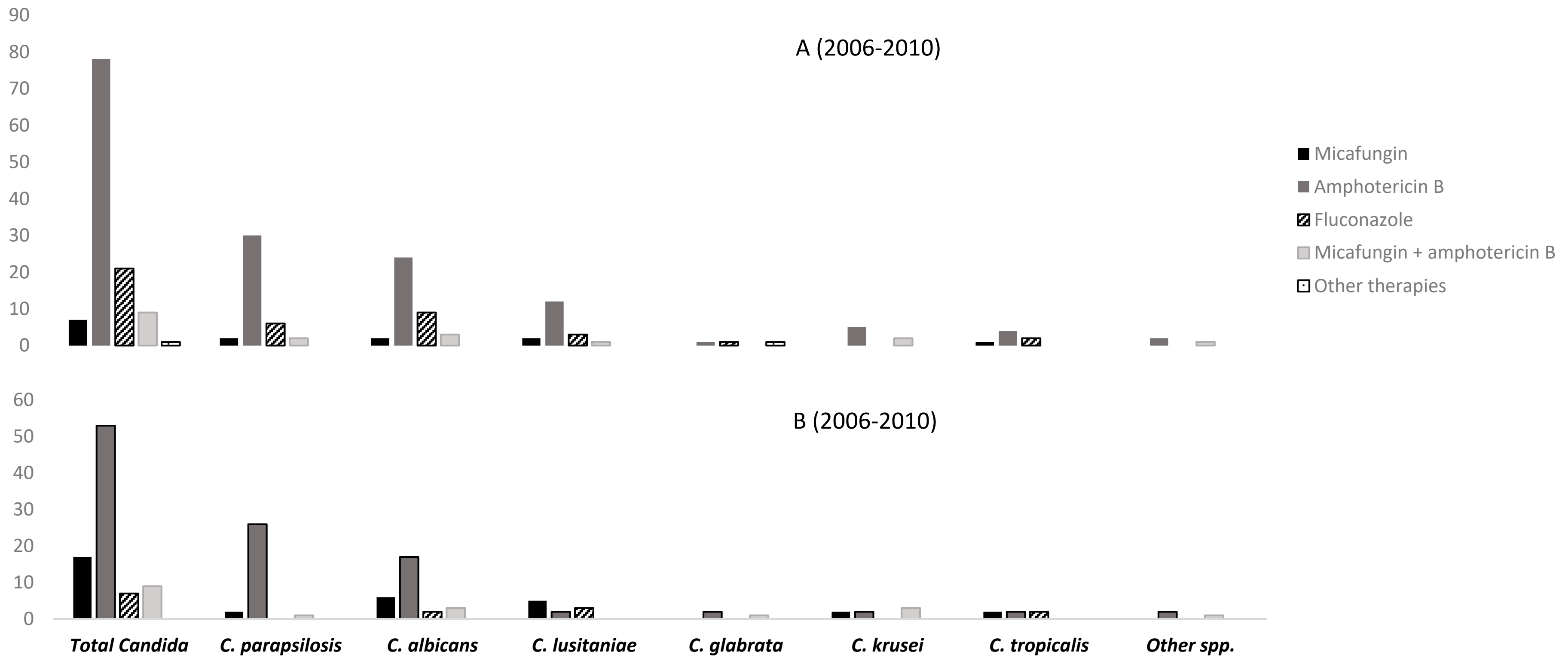

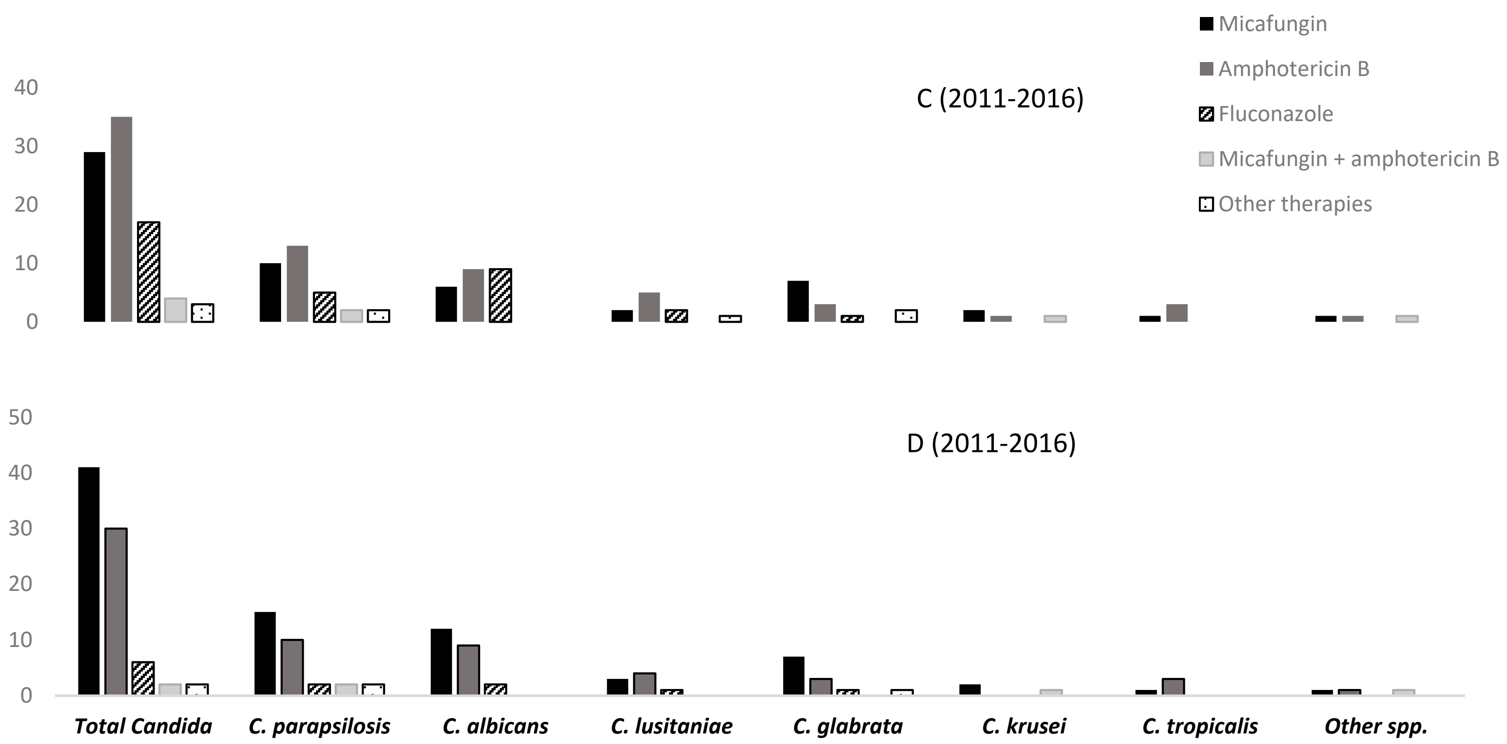

Figure S1. Antifungal agents used for specific Candida species. Agents prescribed by the primary team (A, C) and recommended by the Infectious Diseases team (B, D) from 2006–2010 and from 2011–2016. Antifungal agents prescribed by the primary team (C) and recommended by the Infectious Diseases team (D) from 2011-2016. NOTE. Other agents as initial therapy included micafungin + voriconazole + amphotericin B (1), amphotericin B + fluconazole (2), amphotericin B + voriconazole (1), voriconazole (1), posaconazole (1); as recommended per ID team included amphotericin B + fluconazole (2), amphotericin B + voriconazole (1), micafungin + fluconazole (1), micafungin + posaconazole (1), micafungin + voriconazole + amphotericin B (1).

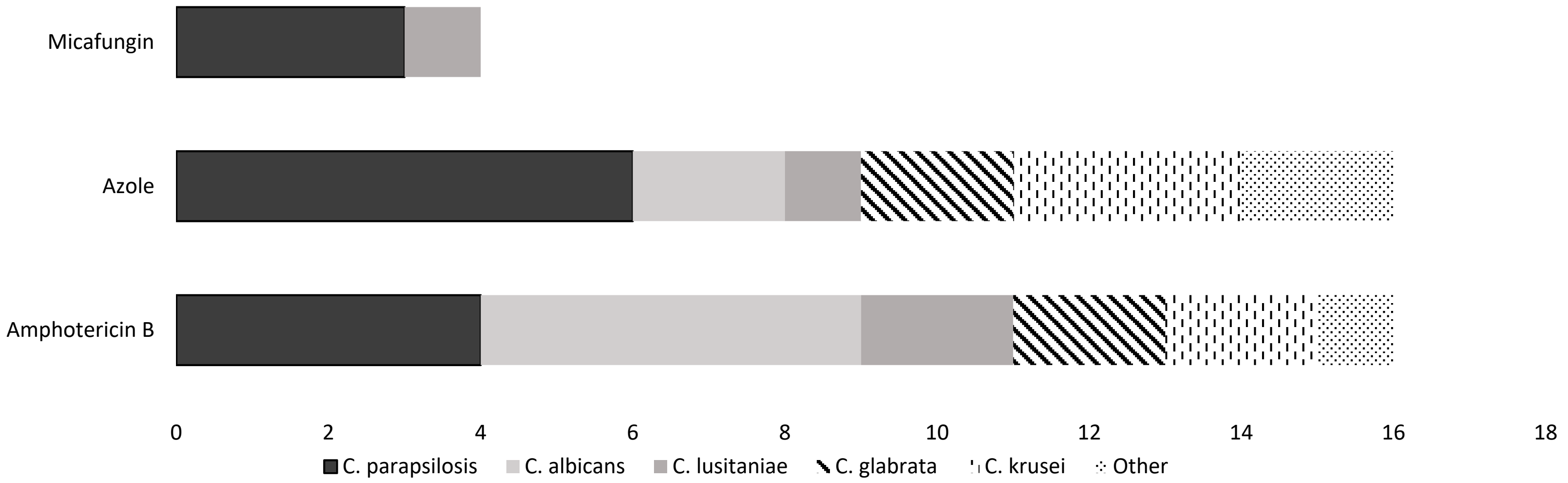

Figure S2. Antifungals received at the time of breakthrough CBSI and species causing breakthrough. Other species were *C. kefyr* while on amphotericin B, and *C. guilliermondii* and *C. membranifaciens* while on azoles.
